# Measurement without management: qualitative evaluation of a voluntary audit & feedback intervention for primary care teams

**DOI:** 10.1186/s12913-019-4226-7

**Published:** 2019-06-24

**Authors:** Daniel J. Wagner, Janet Durbin, Jan Barnsley, Noah M. Ivers

**Affiliations:** 1 Department of Community Health Sciences, Cumming School of Medicine, University of Calgary, 3280 Hospital Drive NW, Calgary, Alberta T2N 4Z6 Canada; 2 Centre for Addiction and Mental Health, 33 Russell Street, Toronto, Ontario M5S 2S1 Canada; 3 Department of Psychiatry, University of Toronto, Toronto, Ontario Canada; 4 Institute of Health Policy, Management and Evaluation, University of Toronto, Suite 425, 155 College Street, Toronto, Ontario M5T 3M6 Canada; 5 Department of Family and Communtiy Medicine, University of Toronto, Toronto, Ontario Canada; 6 Family Practice Health Centre, Institute for Health Systems Solutions and Women’s College Hospital Research Institute, Women’s College Hospital, 76 Grenville Street, Toronto, Ontario M5S 1B2 Canada

**Keywords:** Audit and feedback, Quality improvement, Implementation, Performance measurement, Primary care

## Abstract

**Background:**

The use of clinical performance feedback to support quality improvement (QI) activities is based on the sound rationale that measurement is necessary to improve quality of care. However, concerns persist about the reliability of this strategy, known as Audit and Feedback (A&F) to support QI. If successfully implemented, A&F should reflect an iterative, self-regulating QI process. Whether and how real-world A&F initiatives result in this type of feedback loop are scarcely reported. This study aimed to identify barriers or facilitators to implementation in a team-based primary care context.

**Methods:**

Semi-structured interviews were conducted with key informants from team-based primary care practices in Ontario, Canada. At the time of data collection, practices could have received up to three iterations of the voluntary A&F initiative. Interviews explored whether, how, and why practices used the feedback to guide their QI activities. The Consolidated Framework for Implementation Research was used to code transcripts and the resulting frameworks were analyzed inductively to generate key themes.

**Results:**

Twenty-five individuals representing 18 primary care teams participated in the study. Analysis of how the A&F intervention was used revealed that implementation reflected an incomplete feedback loop. Participation was facilitated by the reliance on an external resource to facilitate the practice audit. The frequency of feedback, concerns with data validity, the design of the feedback report, the resource requirements to participate, and the team relationship were all identified as barriers to implementation of A&F.

**Conclusions:**

The implementation of a real-world, voluntary A&F initiative did not lead to desired QI activities despite substantial investments in performance measurement. In small primary care teams, it may take long periods of time to develop capacity for QI and future evaluations may reveal shifts in the implementation state of the initiative. Findings from the present study demonstrate that the potential mechanism of action of A&F may be deceptively clear; in practice, moving from measurement to action can be complex.

**Electronic supplementary material:**

The online version of this article (10.1186/s12913-019-4226-7) contains supplementary material, which is available to authorized users.

## Background

Audit and Feedback (A&F) is a popular quality improvement (QI) strategy and is synonymous with the terms clinical audit, practice feedback and performance dashboards. Defined as a summary of clinical performance to health care providers over a specified period of time, the popularity of A&F is attributable to the sound rationale that measurement is necessary to improve quality of care [1]. Audit and Feedback seeks to support QI through the systematic assessment of care against explicit criteria and the subsequent implementation of change [2].

It is well understood that a feedback loop reflecting an iterative, self-regulating QI process serves as the mechanism of action for A&F interventions [3, 4]. This feedback loop is composed of three distinct stages: *audit*, *feedback,* and *response*. In the *audit* stage, data are collected (manually or electronically) to capture recent performance for some measure (or series of measures). The audited data are contrasted against some comparator measure such as a consensus benchmark, a summary of peer performance, or historical data of the recipient. In the *feedback* stage, information on the comparative level of performance is delivered to the intended audience. Modes of delivery may include some combination of post, electronic mail, in-person review, or electronic performance dashboard. To initiate the *response* stage, the feedback recipient(s) must i) assess whether a quality of care gap exists, ii) consider whether it warrants a change in effort or in clinical processes or workflows, and then iii) carry out the necessary action(s) to achieve a higher score in the future. Feedback recipients can be expected to weigh their decision in the context of the available resources to act on the gap and on the perceived validity and relative importance of the gap [3, 4]. Following a pre-specified period, the feedback loop would be repeated. Iterations of this feedback loop aim to motivate ongoing efforts to close existing gaps or to identify emergent gaps requiring action. Over multiple iterations, behaviour and/or policy changes by health professionals and organization should address the gaps between ideal and expected care.

For the purposes of this paper, it is important to distinguish between “external” and “internal” A&F efforts. The former refers to initiatives organized, developed, and administered by an entity external to the setting which is the subject of the audit, such as a funder or regional organization. The latter refers to A&F efforts initiated, managed, and maintained by the local healthcare professional recipients of the feedback [5, 6].

While the mechanism of action for A&F is straightforward in principle, the intervention has been described as an unreliable approach to QI [7]. Most prominently, findings from the 2014 Cochrane review on A&F reported a large interquartile range (0.5 to 16%) for improvements in processes of care across a range of clinical conditions and settings [1]. In response, the field has advocated for a shift in inquiry to understand how and when this intervention works best [7, 8]. Efforts to optimize the impact of A&F have emerged, including 15 suggestions by Brehaut et al. [9]. Such suggestions offer guidance to leverage the mechanism of action of A&F by strengthening fidelity to all three stages of the feedback loop. Evaluations that help to identify whether, how, and why initiatives are able to implement fully all stages of the feedback loop may help to unearth additional opportunities to optimize the effectiveness of A&F. For instance, a 2018 study explored the execution of the feedback and response stages in the mental health care context. Failure to execute the latter stage was attributed to unclear expectations and the absence of senior leaders at feedback report meetings [10]. However, a gap persists with respect to evaluations exploring implementation in the context of the entire feedback loop.

The present study aimed to evaluate the implementation of a voluntary, external A&F intervention in a team-based primary care practice context. Specific research questions included: i) To what extent did the implementation of the A&F program reflect a complete feedback loop?; and ii) What were the barriers and facilitators to implementation at each stage of the feedback loop?

## Methods

This study was a component of a larger qualitative investigation by the study team using semi-structured interviews to evaluate a voluntary A&F initiative targeted towards team-based primary care practices in Ontario, Canada. The first study from this investigation explored why primary care clinics chose to participate in the A&F program [11]. The present study evaluated the subsequent implementation of the intervention. Due to considerable methodological overlap, methods and procedures for the present study are summarized here and expanded upon where relevant.

### Setting and context

The Family Health Team (FHT) is a primary-care practice model in Ontario, Canada where a multi-disciplinary team of health care providers collaborate to provide patient-centred care [12, 13]. The FHT model emerged in parallel with the patient-centred medical home (PCMH) and is thought to meet similar standards and requirements [12, 13]. All FHT services are available at no cost to the patient and providers are remunerated by the Ontario Ministry of Health and Long Term Care (MoHLTC) [12, 13]. Team members and roles vary between practices. The multi-disciplinary, non-physician, providers on the team are salaried and may include Nurse Practitioners, Social Workers, Dietitians and other regulated and/or unregulated health professionals. The group of physicians affiliated with a FHT may organize under a range of legal structures and are remunerated under a blended capitation model [14]. It would be appropriate to characterize this setting as a partnership between physicians and the FHT (which represents the interdisciplinary health professionals), as the organizational structures granted to physicians are intended to preserve physician autonomy. The FHT is typically led by an Executive Director who serves as an organizational and administrative lead. Governance of the FHT may be one of three types: i) physician led, where the affiliated physician group makes up the full board; ii) mixed, where governance is shared between the FHT and affiliated physician group; or iii) community sponsored, where governance is led by the FHT and community representatives. At the time of this study there were 184 FHTs in Ontario, serving approximately 3.5 million patients (25.4% of the Ontario population) [15].

### Intervention

In 2014, the Association of Family Health Teams of Ontario (AFHTO) launched Data-2-Decisions (D2D) as an A&F program and strategy to support FHT efforts to measure and improve the quality of team-based primary care [16]. As a not-for-profit advocacy association, AFHTO is mandated to promote the delivery of high-quality primary health care among its membership. AFHTO has engaged in a number of activities to evaluate and subsequently improve D2D as part of a fulsome change management strategy [11]. While AFHTO facilitated recruitment and provided background information for this study, the funding, design, data collection, analysis, and dissemination of this evaluation were independently executed.

D2D is a voluntary initiative, informed by the work of Barbara Starfield to include meaningful measures of quality in primary care [17–19]. For the purposes of this study, D2D is classified as an external A&F initiative since it was organized by AFHTO to provide targeted feedback to FHTs. The measures included in D2D are assessed and selected by an AFHTO sub-committee of relevant stakeholders including FHT staff, beyond Executive Directors, who would ultimately receive the D2D reports. Selected measures were intended to reflect patient centered care, access, and guideline concordant practice. Metrics included within the final category (cancer screening, childhood immunization rates and diabetes care) reflect areas of priority identified by Health Quality Ontario or AFHTO itself as well as Canadian practice guidelines [20–23]. In other words, D2D deliberately consolidated indicators from multiple initiatives and sources believed, from the perspective of AFHTO members, to be of high-priority. An example of the feedback report, the measures of interest, source of data and rationale for inclusion are presented in Additional file 1. At the time of this study, the audit included three sources of data: FHT electronic medical records, annual patient experience surveys, and administrative data. For measures relying on administrative data, values were acquired in their raw format from the agency responsible for housing it or in summarized format extracted from a primary care group practice report produced by Health Quality Ontario [24, 25]. Each participating FHT extracted their measures from the relevant source and then manually transcribed them into an online data-entry portal made available by AFHTO during the audit period. The timing of the inputs to the feedback report, relative to its release is presented in Additional file 1.

Several weeks after the audit process, AFHTO notified participants that their D2D feedback report was available on a password protected website. Notifications were distributed via email, and participants were invited to engage in an AFHTO-hosted webinar to understand the results. As presented in Additional file 1, the D2D feedback report provides a summary of a FHT’s performance relative to a group of peers. Peer status is determined by four self-reported characteristics of each participating FHT: urban or rural setting; teaching status (none, academic, non-academic); access to hospital discharge data; and roster size of the practice.

To facilitate D2D participation, AFHTO collaborates with Quality Improvement Decision Support Specialists (QIDSS). These individuals are funded by the Ontario MoHLTC and are a shared resource among a group of FHTs to support ongoing quality improvement activities. As QIDSS’ responsibilities are exclusive to QI, they are not involved in the daily operations of the FHT. The QIDSS role varies between FHTs to meet individual practice needs. AFHTO provides QIDSS staff with specific training so that they can support D2D. As the availability of the QIDSS resource is not linked to D2D participation, these individuals may engage in separate QI activities within each practice.

### Data collection

The interview sampling frame was drawn from FHTs that met two eligibility criteria: i) the FHT had agreed to participate in an AFHTO-led developmental evaluation of D2D, and ii) the FHT had participated in at least one iteration of the A&F program. Following the release of the third iteration of the feedback report, Executive Directors (ED) from eligible FHTs were invited to participate in semi-structured interviews to discuss their experience with D2D. These leaders were targeted as informants as they were the intended recipients of the feedback report. In recognition of the role played by other team members, additional informants who were more familiar with D2D processes and procedures were invited at the discretion of the ED. Additional informants included Physician Leaders, QIDSS staff, and interdisciplinary health professionals.

Criterion sampling was utilized to ensure variability across FHTs for practice setting, roster size, teaching status and the Standardized Adjusted Clinical Group Morbidity Index (SAMI). The SAMI is a measure indicative of the relative complexity of patients rostered to a FHT [26]. Informants were recruited via email, with the first group of practices identified by AFHTO using an in-house composite measure of quality which is included in the D2D feedback report. All subsequent recruitment groups were selected by the lead investigator (DJW) by actively monitoring representation across the four above measures [11].

As previously described, the Consolidated Framework for Implementation Research (CFIR) was used as the conceptual framework for this study [11, 27, 28]. Two key factors served as the rationale for the use of CFIR against some alternative. First, CFIR reflects a consolidation of theories across implementation science. Second, CFIR offers researchers flexibility in selecting constructs which are considered to be relevant to the study. Furthermore, having a standardized taxonomy of constructs allowed for some comparability of findings across studies [27, 28]. In the present study, it offered a means to evaluate whether, how, and why different types of FHTs may have engaged D2D to guide their QI activities.

The interview guide for the present study was prepared from a template made available by CFIR’s developers. As described previously, the template guide was adapted to reflect the objectives of the present study [11]. Further revisions to question structure and sequencing occurred prior to recruitment following piloting of the interview guide with a FHT leader who did not participate in the study.

Interviews began by defining the study context and building rapport. Open-ended questions were then asked to explore how FHTs use D2D, their motivations for participation, and the resources required to participate. Probing questions were used to further explore areas of interest and specific points raised by informants. Next, participants were invited to complete a usability testing exercise of the online D2D feedback report. Informed by user-centred design methodology, usability testing is a technique to evaluate whether the intended users of a product or service can achieve desired tasks. In the context of the A&F literature, usability of the feedback report is an issue which is addressed infrequently, despite the proliferation of electronic feedback [29–32]. Participants were encouraged to “think out loud” as they attempted to complete two distinct tasks on the D2D website [33]. In the first task, participants were asked to load their team specific results in the interactive feedback report on the D2D web-page. In the second task, participants were asked to review their results for the core D2D measures. This approach offered an opportunity to ask probing questions seeking confirming or disconfirming evidence from earlier in the interviews. For example, in the main interview, participants were asked about D2D support materials. Data collected could be validated in the usability exercise by asking the user where they would look for clarity about the data, among other items. The usability testing supported two distinct outputs. First, a summary was provided to AFHTO to support future enhancements to the D2D website design. Second, data were used to support the analysis for the present study as described in the procedures below.

### Analysis

Qualitative analysis was facilitated by NVivo software [34]. A framework approach was utilized to analyze the transcripts based on constructs within the CFIR. As with the interview guide, a modified version of the codebook, made available by the CFIR developers was used to analyze interview transcripts. Double coding and the development of the codebook followed the procedure previously reported [11]. Construct selection for the frameworks was based on identified relevance to the research questions and was done following data collection, but *prior* to analysis. This approach can be described as a directed content analysis that leveraged a priori constructs from an established framework and is consistent with previous uses of CFIR [35, 36].

The framework for the first research question (whether D2D implementation reflected a complete feedback loop), included the CFIR constructs “Executing” and “Engaging”. Text was then analyzed inductively to classify findings into one of three stages of the feedback loop: *audit*, *feedback*, and *response*.

With respect to the second question (barriers and facilitators to implementation reflective of a complete feedback loop), the framework was generated from thirteen constructs: “Relative Advantage”, “Adaptability”, “Relative Priority”, “Organizational Incentives and Rewards”, “Compatibility”, “Leadership Engagement”, “Available Resources”, “Access to Knowledge and Information”, “Opinion Leaders”, “External Change Agents”, “Reflecting and Evaluating”, “Parallel Initiatives”, and “Usability Testing”. To identify emergent themes from the data, the text was analyzed inductively, and the resulting themes were then categorized by one of three CFIR domains: *intervention characteristics, outer setting,* and *inner setting.*

## Results

Thematic saturation was reached following 18 interviews (25 informants from 18 FHTs). Fourteen participants were Executive Directors, while the remaining eleven were split between physicians, QIDSS, and interdisciplinary health professionals. Details of the eligible and recruited practices, as well as the number and type of informants in each interview are reported in Additional file 2 and additional interview details have been reported elsewhere [11].

### Implementation of the a&F program

A summary of the findings detailing the implementation of the A&F program are presented, along with supporting quotes, in Table 1.

**Table 1:** Summary of Findings of the Implementation of the A&F Program

Theme	Result
Audit	• Led by QIDSS or internal data specialist.
“[QIDSS] comes to us to say, … here’s the list of possible things we can submit, which ones do you want to, which ones do you not want to. We ask her a few more questions about details of how this information is gathered, and then she runs it. She sends it to us first, to say, do these numbers make sense because they don’t always …” (ID = 001)	
Feedback	• QIDSS reviews result, presents to FHT leadership. • Feedback report re-created when distributed.
“I access the data and I prepare a report back to each of the executive directors and pass it onto them. I know that they have shared it with their board in the past, but it’s basically just been given … it’s just been noted that here’s the D2D report from the QIDSS.” (ID = 016)	
Response	• No FHT used D2D for QI. Data used in annual reports. • Attempts to validate feedback results, led to discarding of report.
“At this point, I would say no. There are no actual decisions that are being made as a result of the data. There’s some passing interest in it, but there’s not been any actual … like there’s been no quality improvement exercises because of the data yet.” (ID = 016)	

The *audit* stage was led by a QIDSS, or an internal FHT staff member for those practices without the QIDSS resource. These individuals queried each data source and manually transcribed the values into their respective fields in the D2D data entry form.

For the *feedback* stage, it was generally the responsibility of the QIDSS to review the results and present the findings to FHT leadership. In some cases, the feedback report was viewed directly by the Executive Director or other leaders. Results would then be shared with a quality improvement committee or board of directors, sometimes following modifications to the report to include comparisons to the QIDSS partnership. Further dissemination of the feedback report to specific providers or staff members was limited.

**Fig. 1:**
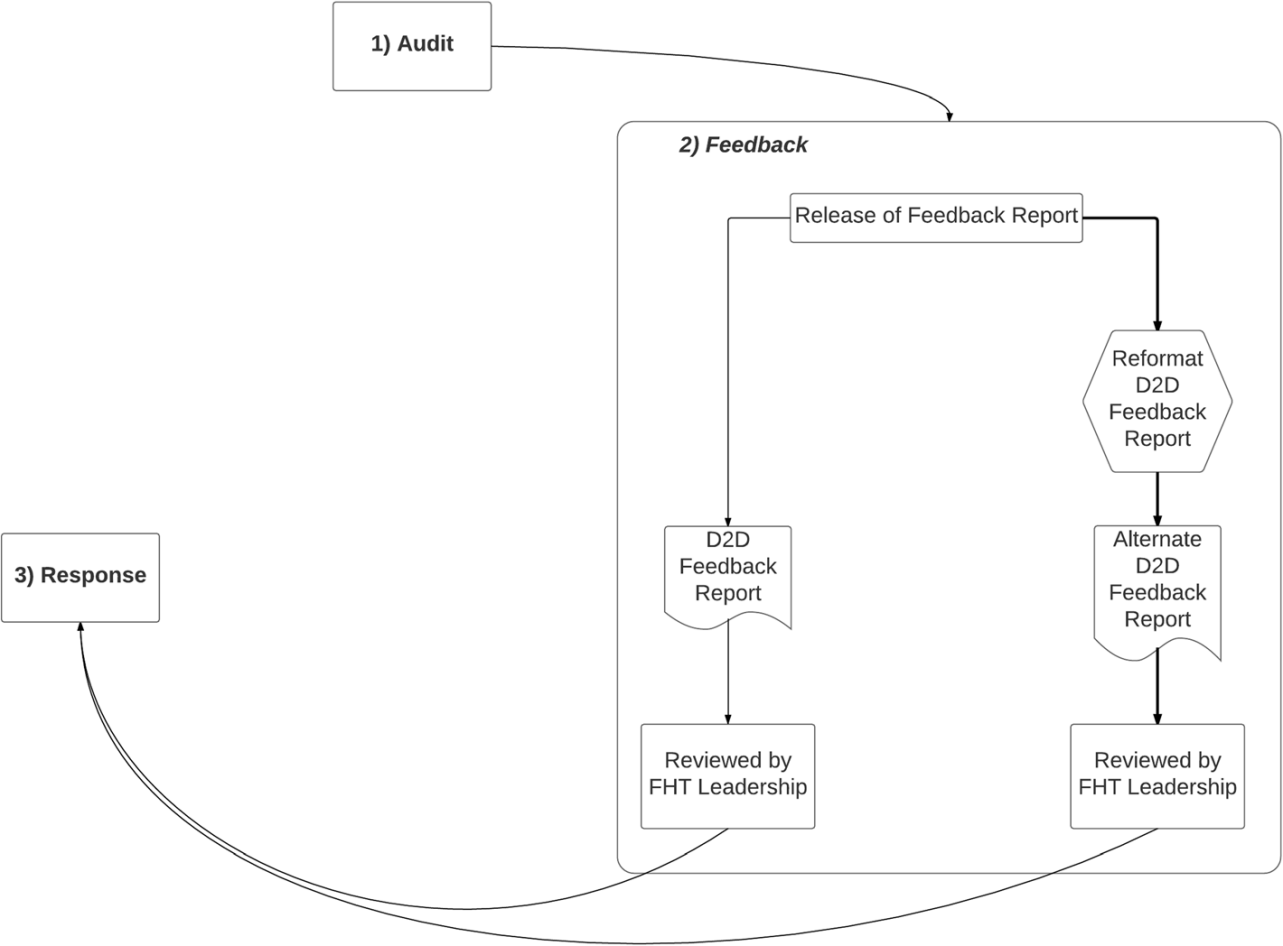
Diagrammatic Summary of the Implementation of the Data-to-Decisions (D2D) Audit and Feedback Program. The figure represents a diagrammatic summary of how interviewed practices implemented the Data-to-Decisions (D2D) audit and feedback program. The observed state of implementation reflected an incomplete feedback loop as characterized by the fact that the response and audit stages were not linked. The *feedback* stage summarizes the observation that the feedback report was sometimes reformatted prior to review by practice leaders

The *response* stage involving the assessment of practice-gaps as well as any subsequent action plans to improve the quality of care was not described by any key informant. Some did report that D2D results were used for narrative purposes in mandatory annual regulatory submissions. Thus, the perceived implementation state of the D2D audit and feedback initiative at the time of this study could be represented by an incomplete feedback loop (Fig. 1).

### Barriers and facilitators to implementation

Several barriers and facilitators were identified as contributing to the implementation state of the D2D initiative. Such factors are presented under the relevant CFIR domain below with a summary of results reported in Table 2. Supporting quotations are included in the corresponding Table following each result.

**Table 2:** Summary of Barriers and Facilitators to Implementation

Result	Impacted Stage of Feedback Loop		
	Audit	Feedback	Response
Intervention Characteristics			
Cycle Frequency. FHTs felt that they did not have enough time to develop or implement QI initiatives between iterations of the feedback report.			B
Data Validity. Measures were insensitive to team behaviour. Data were not reflective of current performance due to duplication from other reports and reliance on administrative data. Technical definitions were unclear to informants.			B
Design. Visualizations were difficult to interpret; the website was hard to navigate and lacked functionality to print or share the feedback report.		B	B
Outer Setting			
QIDSS Dependence. QIDSS were the implementation leaders of D2D, with many practices dependent on this resource.	F	F	B
Inner Setting			
Relative Priority. Participation in other A&F programs influenced the priority of D2D.			B
Resource Requirements. D2D audit was considered to be labour intensive; FHTs lacked staffing to support further implementation.	B		B
Team Relationship. Physicians saw the FHT as an entity to which they were not accountable.			B

Notes: 1. *B* Barrier and *F* Facilitator

#### Intervention characteristics

##### Cycle frequency

Participants cited the frequency of audit cycles as a barrier to implementation. It was specifically noted that the six-month gap between the release of D2D 2.0 and D2D 3.0 was insufficient to observe the effect of any change. Some participants further expressed feedback fatigue. (Table 3).

**Table 3:** Supporting Quotations for “Cycle Frequency”

• Some of the D2D timelines have been very aggressive. Especially the last one, D2D 3.0, it came out very quickly after D2D 2.0. So, even the opportunity to do the decisions part between the two … There was really no time to do it. … All it actually allows you to do is, when the next iteration comes, do a reflection around, what one do we really want to continue to participate in and which ones don’t? (ID =003)	
• In City-X, all the executive directors meet every 6 weeks and we raised it. Someone was going to take it to the [AFHTO] board to say that we felt that the return on investment for the frequency is diminishing rapidly. It’s a very good idea, too many iterations, not well thought out, too. (ID = 013)	
• I thought, for me, it was happening a bit too quickly. I think they could spread it out a little bit more. It felt like D2D 2.0 just happened and then we were getting ready to submit D2D 3.0. (ID = 012)	

##### Data validity

Many FHTs questioned the validity of the data reported in D2D. Poor documentation was cited as a contributor to data validity concerns. Informants suggested that vague definitions in D2D support material did little to address their mistrust in the data. Many did not understand why the complete methodologies for each measure were not easily accessible. In the absence of such documentation, participants were concerned about methodological consistency between practices, limiting the utility of peer comparison. In addition, participants had a range of perspectives regarding the recency of the audit for each measure. A minority of participants recognized that the multi-sourced nature of D2D implied that the measures may not represent similar time periods (as reflected in Additional file 1). Some became aware of this when asked about the relative timing of measures during the usability exercise. (Table 4).

**Table 4:** Supporting Quotations for “Data Validity”

• You know, it’s old data. It’s like a newspaper that’s a year old and picking it up, and reading it. (ID = 002)	
• Some of the indicators that are on D2D are reports we get from HQO. So, we submitted in the fall for … new ICES data, but then they just send the old stuff from the previous time we did D2D, so we were really reporting the same thing as we did the last instance, which really isn’t that beneficial because your numbers are the same. So that’s just a … it’s not really AFHTO’s fault because HQO is the one that prepares the data, but it doesn’t really make any sense. (ID = 006)	
• I’m not sure why they are in D2D if they are already in the QIP because, again, some of it is based on data that isn’t timely. (ID = 003)	
• But how they’re tracking it is not reflective of the spreadsheet that we’ve done and internally tracked our process. And I think some is made of billing codes. Well, only physicians are billing. The billing is different if you’re seeing your patients are coming from long-term care because the codes are different. So our numbers are really not reflective of internally what we’re measuring. (ID = 012) • They’re telling us they want to know what percentage of patients can get same day appointments or can get an appointment with their family physician. So, we scored low, but we have IHPs that work with these family physicians and so they don’t need to go see their doctor if they want to have their blood pressure checked. (ID = 008) • It’s often unclear, that’s part of what the trepidation is. I’ve had the conversation about this particular one, time spent and I’ve asked things like, I don’t know that, whatever your methodology looks like, I don’t know that you’re factoring in patient complexity, in terms of how much time providers need to spend with people, based on their complexities or their patient profile. And people will say, it’s in there, but they can’t show me how it’s in there. And so then, just saying that it’s in there doesn’t mean that I’m going to trust that it’s in there. (ID = 018)	

##### Design

Participants cited frustrations with the design of the feedback website, finding it difficult to interpret as data visualisations did not adhere to standard conventions. For example, while the Effectiveness measures in Additional file 1 are presented on a percentage scale – details about the scale are absent. Participants also sought the ability to export or share their report directly from the website to support quality improvement, a functionality that was not available at the time of this study. Furthermore, participants expressed a desire for a more accessible data dictionary as well as a more sophisticated website where they could interact with a specific indicator to access further information. (Table 5).

**Table 5:** Supporting Quotations for “Design”

• There’s no option to, maybe export it out of the web site into Excel or anything. The graphs, you know, to get to the number you had to move your cursor over so that meant me sitting there writing out all the numbers on a piece of paper and then transferring it to Excel. The expanded data wasn’t on the web site at all, so that was a little bit disappointing, so that meant a lot of work afterwards. And then, I guess, when you went to go click on the targets it brought up a PDF. There was just some disconnect with the whole report on how it come out. So if there was any way to export it so that you could get a two or three page report that you could hand to someone, I think that would be a lot more helpful than a lot of links that didn’t really seem to connect well. (ID = 005)	
• The presentation of D2D could be better, like the way that the website … the logic behind the way that .. like you drill down and stuff into that is different. Most of the other reports are in some sort of chart format, where it’s actually just figures. There are no graphs and stuff like that. So, D2D attempted to visualize a lot of that data, and by doing so, makes it sometimes awkward to understand, which is kind of the reason that I make my own report from it. (ID = 016) • M: Where would you look for the data dictionary? R: But for me to look for anything on the site, it’s easier for me ot go to my computer because the site is not easy to navigate. (ID = 003)	
• I would like to see, I guess it’s just the way that I’ve always learned, is when you have a graph there’s typically a title for the graph and then information. So like effectiveness which is the area where the child, the immunization information is under. It doesn’t really tell me effectiveness of what. It just seems to be kind of out there. (ID = 019)	
• I would have to go into another page, click on colorectal screening, it would take me to the AFHTO page that would tell me where to go for my information. And it usually takes me to HQO or something, some long report, or actually an AFHTO page. You have to drill, drill, drill, drill down. It would be great if you could hover over this and it would say, this is this. It’s a lot of work to be able to figure that out. Like, I can’t remember whether it’s over 50, or over 65, or over this, that you know, the population and the exact, you know, numbers that you need to be able to put that in off the top of my head. So if I was presenting it to a group I would do all that pre-work ahead of time, I would go and print those page, or make sure it’s off the top of my head. (ID = 002).	

#### Outer setting

##### Dependence on QIDSS

The availability of the QIDSS served as a facilitator to implementation. While there were a select number of practices where the QIDSS was not needed, this was limited to FHTs with in-house expertise to fulfill QIDSS-like responsibilities. In all other cases, a FHT’s participation in D2D was dependent on this resource. However, participants noted that at the time of data collection for this study, implementation support did not extend to the *response* stage of the feedback loop. (Table 6).

**Table 6:** Supporting Quotations for “Dependence on QIDSS”

• I think it would fall apart if the QIDSS were not there. I think D2D would totally fall apart in the province. Even if we built an infrastructure where you can manage it internally, you do need somebody that can push the agenda, because in your day-to-day business, you’re going to put this further and further down the priority list. This is all faith coming to the table to participate. (ID = 003).	
• M: Does your FHT have sufficient resources to implement D2D? R: With our QIDSS specialist, yes. M: That QIDSS specialist, that person would probably be like the minimum required resource needed. R: Oh, yeah, if we didn’t have him we wouldn’t be able to do it. M: Is it the knowledge that that person brings, the skill-set? R: Yes, and the time. (ID = 008)	
• M: So, you did not participant in the recent release, which was done last month? R: No, because the timing of our new QIDSS position, it didn’t work out when Name-X started to actually be able to submit everything. So, the plan is to get ready, now that he’s been here for a while, to do 4.0 this fall. (ID = 009)	
• M: Do the FHTs you work with have sufficient resources to participate in D2D? R: Yes. M: Would those resources be you? R: Yes. M: So, if you had to step away … R: They would stop reporting. I shouldn’t say that. One would probably still report, the small FHT, that executive director. There’s where I have the QIP committee. So, one of them would, but the other two wouldn’t. (ID = 016).	

#### Inner setting

##### Relative priority

FHTs had access to a wide array of A&F initiatives and participation in these varied widely. All FHTs participated in mandatory quality improvement programs, which included the Quality Improvement Plan (QIP) and patient experience surveys, as outlined in provincial legislation [37]. Participants had access to feedback reports covering a range of indicators from several existing provincial initiatives [24, 38, 39]. The most common were Health Quality Ontario’s (HQO) Primary Care Group Practice Report; the Screening Activity Report (SAR) from Cancer Care Ontario (CCO); the Electronic Medical Record Administrative Data Linked Database (EMRALD); and the Canadian Primary Care Sentinel Surveillance Network (CPCSSN) from the College of Family Physicians of Canada [38]. FHTs also conducted their own internal A&F programs with varying degrees of sophistication. Often, D2D was viewed to be of lower priority relative to these other A&F initiatives. Relative priority was a function of regulatory requirements, available capacity and maturity to support QI, and the perceived attributes of each A&F effort. Specifically, FHTs which lacked internal reporting mechanisms, or which did not participate in external programs assigned greater priority to D2D. (Table 7).

**Table 7:** Supporting Quotations for “Relative Priority”

• We rely on our patient surveys and our diabetes stats quite highly, because they are monthly, so they’re real time, and because with our diabetes stats we can then drill down into those stats and find out who the ones are that we’re missing. So that’s very concrete for us. Some of the HQO and the D2D stuff is more higher level. (ID = 015)	
• Other problem is we have all of our information, which is second to none through CCPSN and UTOPIAN. We now have shared data. We’re housed with the hospital, where we can look at acute and primary and see how we’re going in that area. This is a bit of a make work project for us, but we participate because we thought we’d be very useful to be part of the bigger picture. (ID = 007)	
• I think what I meant to let you know is that we try to speak to the quality committee this time for 3.0 prior to the board meeting, but it didn’t quite make it through the agenda. And so, the board got presented first, and hopefully we’ll have time to talk to the quality committee about it next time. But our priority was the QIP that’s due out on April 1st, and we had a lot of discussions around that as a priority as opposed to the dissemination of these results. (ID = 002)	
• I think that we’ve spent quite a lot more time and got more value out of the Cancer Care Ontario SAR Report than anything else. (ID = 014)	

##### Resource requirements

Participants noted that the D2D audit was labour intensive and that a certain level of human resources was necessary to facilitate the implementation of D2D. Furthermore, that the QIDSS were focused on D2D left participants concerned that there was little capacity for this resource to support other QI activities. This barrier to implementation was not observed among FHTs with dedicated, internal quality improvement support staff, who could support data management, analysis and facilitation of quality improvement. (Table 8).

**Table 8:** Supporting Quotations for “Resource Requirements”

• … my QIDSS person spends a lot of time giving D2D data. (ID = 001)	
• With D2D there’s more work involved, and we’re trying to minimize that, but there certainly is more work involved. (ID = 014) • If they keep doing it at the rate that it’s going right now, I’ll probably not participate. I’ll probably talk to our group about not participating and having our quality improvement data support specialist do something else, because that is really all she has been doing, is getting it ready and doing these submissions. (ID = 013)	
• I don’t think that, from a team perspective, the teams have the time and capacity to attend to this to the level that perhaps HQO thinks we should. So it’s one thing to say you have the staff resources in a QIDSS specialist to assist … But when you look at how people are spending their time in organizations, and you ask them to engage in D2D or other quality improvement initiatives, it takes time, people attend meetings … there’s a whole bunch of pieces. When they’re doing that, they’re not seeing patients. And so, the quality improvement initiative is, the Ministry going back to your thing around the policy climate, is really trying to drive increased accessibility. When I have 10 people in a meeting for two hours to talk about quality improvement, that means they’re not seeing patients, which reduces access of our patients to our team. So, in terms of resources, there needs to be greater organizational capacity to be able to plan, develop, and implement quality improvement initiatives beyond a part-time QIDSS specialist. Otherwise, we really are working in conflict in terms of trying to give patients greater access to our providers, while, at the same time, distracting our providers by trying to engage them in things like quality improvement initiatives or other things that seem to come down from the Ministry within that particular policy climate. (ID = 005)	
• Because it’s difficult to extract information from the EMR our HPs and our RN’s have to spend a lot of time extracting this data. I think there is value in it because I think we have to demonstrate … so, it’s just that it’s hard to pull the data and we don’t have a quality improvement person. So, it’s taking time away from patient care. (ID = 013)	
• I think now as a QIDSS, being a resource myself, I think it’s enough that I’m able to collect the data and submit on it but being able to act and implement those changes that are required to lead quality improvement, there definitely needs to be some more resources put in place, especially if it continues to grow. One person can only do so much with the time so that face time I have at each FHT and the influence I have, there really needs to be more of us I would say. (ID = 014)	
• R1: Our data person is a dietician. R2: Yes. She is a half-time dietician, half-time data person. R1: She was never a data-person. We just gave her the job and she learned it on the job, which was great. I think that is also one of the concerns, too. From a knowledge transfer perspective, if she left tomorrow, we would be in a lot of trouble. (ID = 003)	
• And, we have seen with D2D, we were usually at the top. But, we also have a lot of resources to help us get to that top. So, some of our comparators do not have any data managers gleaning their data. They don’t have all of that. (ID = 007)	

##### Team relationships

Implementation was also affected by the relationship between the physicians and the rest of the team (i.e., the executives and allied health professionals of the FHT). Participants noted that some physicians saw the FHT as an entity to which they are not accountable. Informants cited difficulties in engaging physicians who were close to retirement and/or who practiced in other care settings (ie. emergency departments) (Table 9).

**Table 9:** Support Quotations for “Team Relationships”

• … The organisation is made up of two teams, the FHT team and the FHO team, and the FHT team is very, very separate from the FHO team. If I could go back in time, I would try to figure out a way to set up the structure that I wasn’t an employee of the FHT, that I was an employee of the FHO, and that would be my angle, you know what I mean, that I actually work for the doctors? (ID = 016)	
• I think with our providers being an independent FHO, they don’t always see how this affects them. It doesn’t affect funding, it doesn’t affect the amount of allied health professionals that you have, it has no concrete affect on their practice, other than whether patients are happy or not. (ID = 015)	
• And we don’t have to get two doctors to agree on anything to actually make it happen because it’s the community-based nature of this. So decisions are really made taking into account how it affects not just the doctors, but the Its and everyone else. Really the decisions about going along with D2D were really mostly determined by the direction that Name-X thought we should take and then passed down rather than the other way around. (ID = 014)	
• Huge changes would be more difficult just because we’re not really allowed to tell them how to work. Basically we have to try it with one physician and then say, hey, you know what, this worked really well, look at the difference in his numbers from doing this for a couple of months. And even so, it’s the same physician every time, so I think some of the doctors get a little, well, I don’t want to hear that from him anymore. We actually do have three physicians on our quality committee now which is great because I think they’ll be willing to try more things too spread them to the physicians they work with. So we have multiple sites of physicians so that makes it difficult too. (ID = 006)	
• We have a number of physicians who are closer to retirement or slowing down or getting out of their practices. They just don’t really have the enthusiasm to implement changes or to try to do something in a different way, whether it’s changing the way that they report something in the EMR to like I’ve been doing it by paper for 30 years of my life. And tehn I finally converted to the EMR eight years ago and you’re not going to tell me how to do something different for two years before I retire. And then the other factor is just time. As I mentioned earlier, many of our physicians they work in the Emergency Department for a smaller community hospital. So they work in the ED, they’re working on the floors, they’re seeing patients, they’re working in long-term care and they just don’t have the time. They have their own admin time to work on or they have their own clinic time where they have to be here doing that, so that’s kind of one of the struggles. And some individuals see the family health team as being the family health team and the physician group being the physician group and we work together, but we don’t have to play together kind of thing. (ID = 019)	

## Discussion

This study evaluated the implementation of a voluntary, external A&F initiative, known as D2D, for primary care teams. At the time of evaluation, implementation of D2D reflected an incomplete feedback loop, as respondents indicated no action following the response stage of the A&F cycle. In other words, during the first 16 months of implementation the D2D initiative did not yet reflect an iterative, self-regulating process that directly supported QI. Barriers to implementation emerged as a result of data validity concerns, labour scarcity, the dependence on an external implementation champion, and the practice structure.

### Comparison with previous literature

Several independent findings are consistent with previous literature on A&F and quality improvement in primary care. For example, there is consistent evidence suggesting that developers of A&F interventions should anticipate that feedback recipients will question the validity of the data. In addition to delays between the time of measurement and reporting, common concerns include inadequate scope of measurement, indicators restricted to physician (rather than team) activity, and comparability between peer groups of practices [40–44]. Tensions persist between primary care practices and other levels of the health care system in prioritizing measures of quality. Practices must balance resources committed to improve metrics in A&F with other quality of care issues [41]. Over multiple feedback iterations, recipients may re-evaluate their validity concerns.

Additional research has identified that feedback uptake can be enhanced through the provision of in-person facilitated feedback, through respectful relationships between providers and recipients [45]. Unfortunately, the present study’s findings reveal that the intervention characteristics of D2D failed to provide opportunity for such discussions. Therefore, the identification of best practices to engender faith in the data is an important area of future research.

A second issue is the resource trade-offs that practices must make between a specific A&F effort, other QI efforts and general practice administration [43, 46]. Previous research has demonstrated a relationship between practice size and experience in using A&F to support QI. Findings have consistently shown that restrictions to labor or experience can limit the capabilities of a practice to implement relevant audits or leverage the feedback loop appropriately [41, 43]. As a result, practices consistently outsource this work as was the case with the present study’s finding that D2D implementation was dependent on an external resource (the QIDSS).

The outsourcing of QI processes is not a new phenomenon in the FHT setting. Previous research by Kotecha et al. explored how external practice-facilitators supported FHTs in their QI planning processes. While these external resources served to coach the FHTs, the practices expressed a clear preference for these agents to lead such activity [46]. The consistent labour scarcity and alternative priorities may lead some to conclude that dedicated funding to outsource QI efforts should be continued practice. However, such policy should be treated with caution. An area for further research is exploring whether and how team-based practices could be encouraged to develop QI leadership and skills internally rather than seek to outsource them.

A third issue is that A&F interventions are rarely evaluated in the context of their mechanism of action. An exception is the work by Pedersen et al. which, like the present study, evaluated an A&F initiative which failed at the *response* stage [10]. The present study offers additional insight towards understanding the inconsistent performance of A&F – the incomplete feedback loop. This implementation state was a function of an interaction between the selection of data for audit (Additional file 1), the available resources of a participating practice, and the design of the feedback report (Additional file 1). Further complicating matters is the tenuous relationship between the physician group and the rest of the primary care team in the FHTs that participated in this study. These contextual factors mean that enhancements of the intervention characteristics may not enhance the intervention’s implementation (fidelity to a complete feedback loop) – or its ability to improve quality of care. Future research is necessary to better understand the extent of this problem in the context of various team-based primary care models and performance feedback initiatives.

### Adherence to optimal feedback recommendations

An assessment of the present study’s results in the context of Brehaut et al.’s 15 suggestions for effective practice feedback optimization offer insight into the mechanics of the feedback loop. D2D featured many strengths that enabled its uptake [11]. First, D2D was supported by the credibility of a trusted source as the initiative was led by a team-based primary care advocacy organization [9]. As reported previously, a strong motivator for participation in D2D was the underlying intent to create an A&F report reflective of team-based primary care which would grow into a best-in-class initiative [11]. For many FHTs, participation in the *audit* and *feedback* stages may have been in service of this goal and not quality improvement [11].

Second, unlike many A&F interventions studied [8, 47], D2D included multiple instances of feedback which should have enabled the intervention’s mechanism of action [9]. The intent of this feature is to leverage the construct of observability, one of several key concepts in diffusion of innovation theory [48]. In delivering three iterations of feedback over a 16-month period, recipients should have had some sense of the intervention’s ability to impact QI. However, cycle frequency was described by participants as a barrier to implementation, with some informants complaining of audit fatigue. Given that repeated cycles of D2D did not yield a complete feedback loop one can conclude that the criterion for multiple instances of feedback should be considered necessary but insufficient for A&F to support QI. Concerns regarding data validity and the design of the feedback report overwhelmed the potential advantages from observability by limiting participants’ engagement in the *response* stage. This demonstrates the need for A&F developers to carefully consider whether quality measures of interest can be accurately and rapidly measured and reported in a digestible format, at a frequency to enable observability.

Several additional characteristics across the *audit*, *feedback* and *response* stages may have contributed to the incomplete feedback loop [9]. For example, D2D included general data (rather than specific detail actionable by the recipient), using a single mode of feedback delivery (rather than multi-modal), without supports perceived as adequate to enable action. A summary of the relative timing of the inputs for three iterations of D2D is presented in Additional file 1. While the feedback report had a visually appealing design, users had difficulty interpreting results and accessing support documentation.

### Implications for quality improvement

Both the recency of the data in the audit and the design of the feedback report have important implications for quality improvement. These are captured in the findings detailing that: i) D2D was labour intensive; and ii) acceptance of the feedback was a function of a FHT’s internal maturity with quality improvement. In light of these factors, practices with less quality improvement experience and expertise are provided the impression that the intervention’s feedback loop is complete and is capable of supporting their quality improvement goals. In other words, practices clearly viewed D2D as a complete QI tool and assumed that participation in the *audit* and *feedback* stages were enabling their efforts to develop QI capacity. However, such engagement may reflect nothing more than a quality mirage.

This phenomenon raises several important points. First, some of the 15 suggestions from Brehaut et al. may have greater weight than others as enablers of the mechanism of action for A&F. Suggestions likely necessary (but potentially insufficient) to promote QI which require additional emphasis include: the provision of multiple instances of feedback, the provision of feedback as soon as possible (reflective of current performance), and addressing credibility of the information [9]. Second, progressively encouraging responsiveness by A&F recipients around the full loop may need more attention given that the ultimate aim for many A&F initiatives, including D2D, is to support change management. Third, it may not be desirable to distract health professionals or organizations with quality measurements when the feedback is not actionable or when the supports or resources to engage in the *response* stage are not available. As highlighted by Fixsen et al., ongoing coaching is a core driver of practice change [49]. Performance feedback is important, but the present study illustrates that it may be insufficient to promote QI. The proliferation of measurement detached from actual improvement work is a major risk [50]. Moreover, without thoughtful reflection on underlying causes and systemic solutions, quality measurement can miss the forest for the trees [51].

### Limitations

Many limitations of the present study have been previously reported [11], as the data collection and analysis were completed in parallel. First, as all interviews were conducted within a homogeneous practice setting (Family Health Teams in Ontario) findings may not be generalizable to other contexts. Different practice models may yield different results in understanding the barriers and facilitators to the implementation of voluntary, external A&F initiatives such as D2D. Second, while double-coding was applied to the analysis of certain interview transcripts, thematic coding was completed in the absence of a validation procedure by the lead investigator (DJW). The impact of this methodological choice on the results was limited by the use of deductive coding (i.e., CFIR). It is acknowledged that this approach introduces the risk that some aspects of implementation may be overlooked. All authors agreed that this risk was acceptable in the context of the many strengths derived from the use of the CFIR as the theoretical framework for this study. Not only is the CFIR thought to be a comprehensive, well-established and well evidenced framework – it also promotes knowledge translation through the application of consistent terminology in implementation research.

Lastly, the present findings may not reflect the current implementation state of the D2D A&F program. The program has undergone continued development since the data for this manuscript were collected. In Ontario, quality measurement and management is not yet the norm in the primary care setting and time may be needed to acculturate these approaches [52]. This may be enabled if the multiple agencies conducting A&F initiatives in primary care in Ontario could collaborate [38]. As the present study reflects a cross-sectional assessment of implementation, opportunities for future research should not overlook the application of longitudinal methods to track efforts to reduce barriers to implementation.

## Conclusions

Despite its popularity, A&F remains an unreliable quality improvement strategy. While efforts to understand how and when it works best are ongoing, few studies evaluate the implementation of such interventions in the context of its mechanism of action. This study identified that the implementation of one particular A&F initiative reflected an incomplete feedback loop. Barriers to implementation were attributable to specific design choices which interacted with resource constraints and a dependency on an implementation champion. Substantial efforts invested in quality assessment were unlinked to subsequent action to change processes of care. If the goal of A&F is to promote QI (or to prioritize QI activities in areas of greatest need), it is necessary to consider minimum requirements for both the intervention *and* the recipients’ capacity to respond. Such an achievement depends on alignment and coordinated efforts between health care practitioners and external organizations regarding the outcomes to be measured and the needs of practices to improve on those measures. The deployment of A&F initiatives which effectively leverage the complete feedback loop will enable providers to achieve what they set out to: better care and improved health.

## Additional files

Additional file 1: The Supplemental File is composed of three elements. First, a screenshot of the performance feedback report is provided. Second, a Gantt chart is displayed to document the relative timing of the data included in the three iterations of the feedback report which had been distributed at the time of qualitative data collection. Due to unspecific documentation, date data for indicators sourced from Electronic Medical Records are not reported in the Gantt chart. It is suspected that EMR queries likely varied by practice base and were not standardized to a specific date. The “Admin Cost Data” row is each facet is meant to reflect only cost data obtained from administrative sources. In facets where this field is blank, the cost data are incorporated into the “Admin” field. Third, a table is presented summarizing the source, operationalized definition and the stated rationale for inclusion in the performance feedback report. Data and information summarized in the Gantt chart and the table were sourced from materials provided to the research team by AFHTO. (PDF 565 kb)

Additional file 2: The Supplemental File contains a table summarizing the FHT practice characteristics as well as interview formats and participants for the present study. (PDF 102 kb)

## Data Availability

The interview transcripts analyzed for the present study are available from the corresponding author, pending approval by the Human Research Ethics Office at the University of Toronto. In lieu of complete transcripts, the framework tables used in this analysis may be made available on reasonable request.

## Abbreviations

A&F: Audit and Feedback
AFHTO: Association of Family Health Teams of Ontario
CCO: Cancer Care Ontario
CFIR: Consolidated Framework for Implementation Research
CPCSSN: Canadian Primary Care Sentinel Surveillance Network
D2D: Data-2-Decisions
EMRALD: Electronic Medical Record Administrative Linked Database
FHO: Family Health Organization
FHT: Family Health Team
MoHLTC: Ministry of Health and Long Term Care
NICE: National Institute for Health and Care Excellence
PCMH: Patient-Centred Medical Home
QI: Quality Improvement
QIDSS: Quality Improvement Decision Support Specialist
QIP: Quality Improvement Plan
SAMI: Standardized Adjusted Clinical Group Morbidity Index
SAR: Screening Activity Report
