# Supplementary material for: Measurement without management: qualitative evaluation of a voluntary audit & feedback intervention for primary care teams
Source: BMC Health Serv Res. 2019 Jun 24;19:419. doi: 10.1186/s12913-019-4226-7 (PMC6591867; doi:10.1186/s12913-019-4226-7)
Supplement: Supplementary file 1 — The Supplemental File is composed of three elements. First, a screenshot of the performance feedback report is provided. Second, a Gantt chart is displayed to document the relative timing of the data included in the three iterations of the feedback report which had been distributed at the time of qualitative data collection. Due to unspecific documentation, date data for indicators sourced from Electronic Medical Records are not reported in the Gantt chart. It is suspected that EMR queries likely varied by practice base and were not standardized to a specific date. The “Admin Cost Data” row is each facet is meant to reflect only cost data obtained from administrative sources. In facets where this field is blank, the cost data are incorporated into the “Admin” field. Third, a table is presented summarizing the source, operationalized definition and the stated rationale for inclusion in the performance feedback report. Data and information summarized in the Gantt chart and the table were sourced from materials provided to the research team by AFHTO. (PDF 565 kb) [file 12913_2019_4226_MOESM1_ESM.pdf]

Overview of the Data-to-Decisions Feedback Report

Supplemental File

Feedback Report

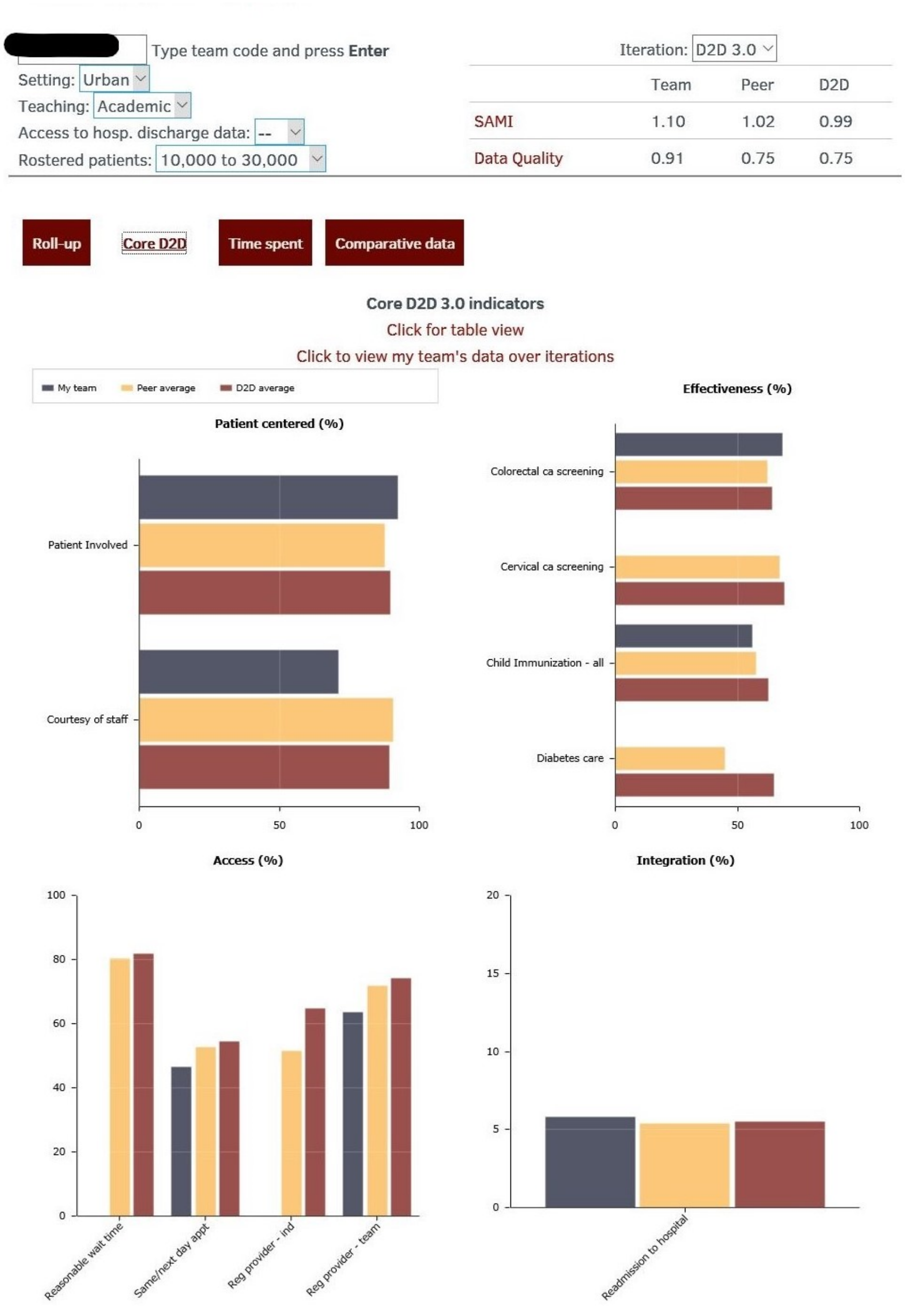

The above figure presents a screenshot of the Data-to-Decisions (D2D) feedback report at the time of the study. A description of each measure is provided in the table below. Data reported in the table were sourced from the Data Dictionary for the third iteration of the D2D feedback report.

Timelines

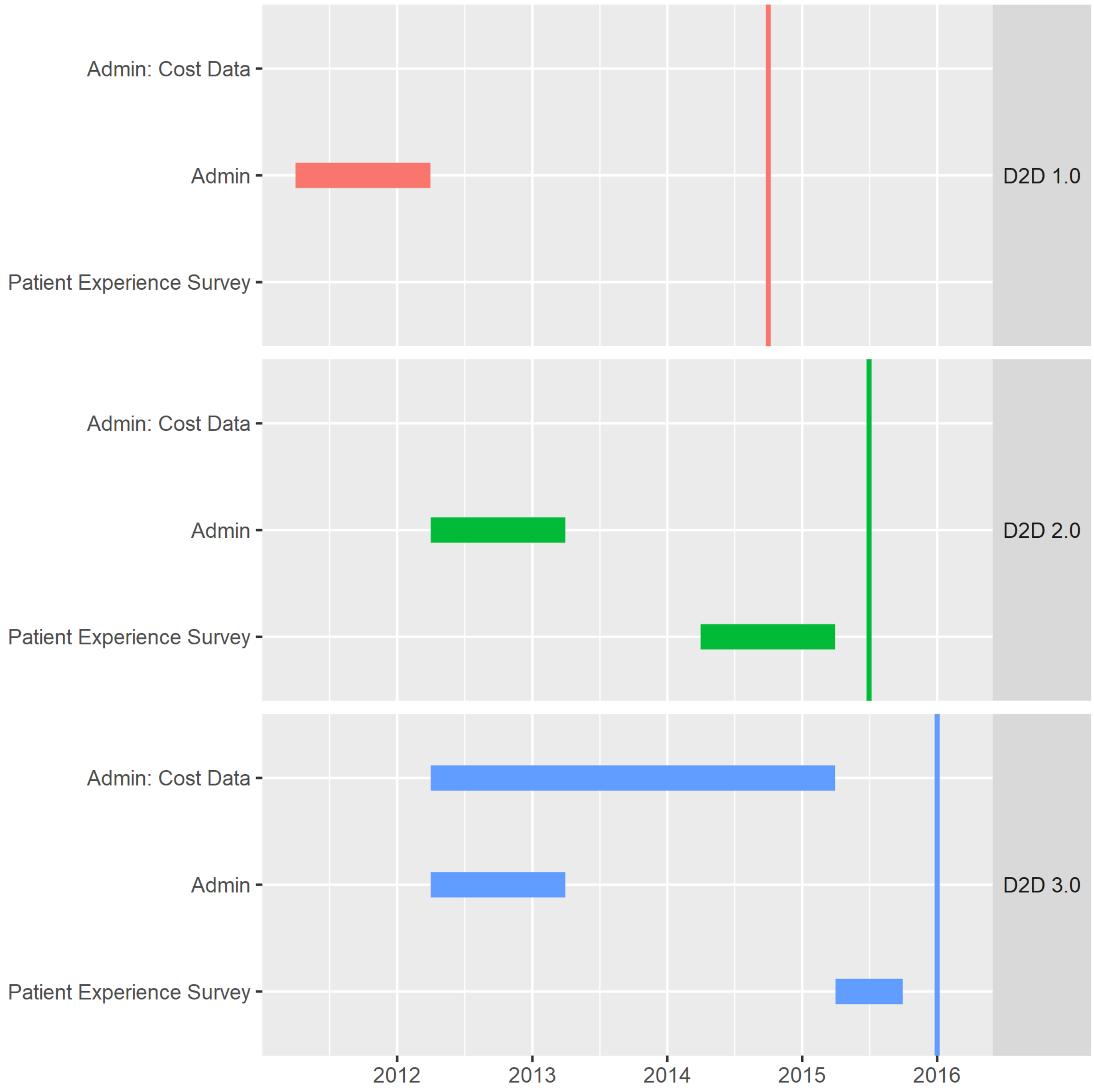

The above is a Gantt chart displaying the relative timing of the data reported in D2D against the release date of each iteration of feedback, as represented by the vertical line. Each facet grouping represents the available data for each iteration of the D2D feedback report. Data were obtained from materials provided to the research team by AFHTO. Date data for indicators sourced from Electronic Medical Records are not reported due to unspecific documentation. It is suspected that queries likely varied by practice according to when the searches were performed to extract the relevant indicators for these iterations of D2D. Lastly, the "Admin: Cost Data" field is meant to reflect only cost data obtained from administrative sources. In facets where this field is blank, the cost data are incorporated into the "Admin" field.

Table 1: Summary of Core D2D Metrics

Measure	Source	Description	Rationale
Patient Involved	Patient Experience Survey	Percentage of patients who report feeling involved as they want in decisions about their care.	Measurement an illustration of respect for patients' and families' values, culture, needs and goals.
Courtesy of Staff	Patient Experience Survey	Percentage of patients who report feeling satified with office staff.	Measure of interest by AFHTO Indicator Selection Committee.
Colorectal Cancer Screening	HQO Primary Care Practice Report	Percentage of patients aged 52 to 74 years old with a fecal occult blood test (FOBT) within the past two years, other investigations within 5 years or a colonoscopy within the past 10 years	Screening and management of risk factors for cancer.
Cervical Cancer Screening	HQO Primary Care Practice Reprot	Percentage of female patients aged 23 to 69 who had a Papanicolaou (Pap) smear within the past three years	Screening and management of risk factors for cancer.
Child Im- munization - all	Electronic Medical Records	Percentage of patients 30 to 42 months (inclusive) who have received all of the ministry-supplied immunizations as recommended by the National Advisory Committee on Immunization.	A measure reflecting care for children, while most others are focused on adults.
Diabetes Care	Electronic Medical Records	Composite indicator based on the percentage of diabetic patients with appropriate perforamnce for at least one of the following indicators: HbA1C testing, HbA1C level, and blood pressure level	Measure of interest by AFHTO Indicator Selection Committee, based on diabetes care as a priority to advance improvement of primary care across participating FHTs.
Reasonable Wait Time	Patient Experience Survey	Percentage of patients who report they were able to get an appointment within a reasonable amount of time.	Measure of interest by AFHTO Indicator Selection Committee reflecting access to care.
Same/Next Day Appt	Patient Experience Survey	Percentage of patients who report they had an appointment on the same or next day.	Measure of interest by AFHTO Indicator Selection Committee reflecting access at regular place of care.
Reg Provider - Ind	Administrati Data	Percentage of Primary care visits for a core service that are made to the physician to whom the patient is rostered or virtually rostered.	Reflects continuity of care with a primary care physician as well as access to care.
Reg Proivder - Team	Administrati Data	Percentage of Primary care visits for a core service, that are made to a physician that belong to the same team as the physician to whom the patient is rostered or virtually rostered.	Reflects continuity of care with a primary care team and is a measure of access.
Readmission to Hospital	Administrati Data	Percentage of hospital readmissions (within 30 days) of admitted patients.	Measure of interest by AFHTO and Health Quality Ontario reflecting health service utilization.
