# Supplementary material for: Measurement without management: qualitative evaluation of a voluntary audit & feedback intervention for primary care teams
Source: BMC Health Serv Res. 2019 Jun 24;19:419. doi: 10.1186/s12913-019-4226-7 (PMC6591867; doi:10.1186/s12913-019-4226-7)
Supplement: Supplementary file 2 — The Supplemental File contains a table summarizing the FHT practice characteristics as well as interview formats and participants for the present study. (PDF 102 kb) [file 12913_2019_4226_MOESM2_ESM.pdf]

Supplemental File 2

Summary of Family Health Team Characteristics and Interview Formats

	**FHT Characteristics**			
	**Total**	**Recruited**	**Not Interviewed**	**Interviewed**
Sample Size	118	45	101	17[1]
	**Mean (SD)**	**Mean (SD)**	**Mean (SD)**	**Mean (SD)**
Roster Size	20,788 (32,429.3)	19,417 (17,383.3)	20,994 (34,644.8)	19,622 (15,126.5)
SAMI[2]	0.99 (0.1)	0.99 (0.1)	0.99 (0.1)	0.99 (0.1)
	**% (n)**	**% (n)**	**% (n)**	**% (n)**
Setting				
Rural	47 (56)	42 (19)	50 (51)	29 (5)
Urban	53 (62)	58 (26)	50 (50)	71 (12)
Hosp. Discharge Data[3]	61 (72)	69 (31)	59 (60)	71 (12)
Teaching Status				
Academic	17 (20)	9 (4)	18 (18)	12 (2)
Non-Teaching	26 (31)	33 (15)	24 (24)	41 (7)
Teaching	57 (67)	58 (26)	58 (59)	47 (8)
	**Interview Formats**			
	**1-on-1**	**2-on-1**	**3-on-1**	**Total**
	**% (n)**	**% (n)**	**% (n)**	**% (n)**
By Practice	66.7 (12)	27.8 (5)	5.6 (1)	100 (18)
Participant Type				
ED[4]	71.4 (10)	21.4 (3)	7.1 (1)	100 (14)
MD[5]	0.0 (0)	66.7 (2)	33.3 (1)	100 (3)
QIDSS[6]	25.0 (1)	50.0 (2)	25.0 (1)	100 (4)
IHP[7]	0.0 (0)	100 (1)	0.0 (0)	100 (1)
Other	33.3 (1)	66.7 (2)	0.0 (0)	100 (3)
Total	48.0 (12)	40.0 (10)	12.0 (3)	100 (25)

Reproduced from:

Wagner DJ, Durbin J, Barnsley J, Ivers NM. Beyond quality improvement: exploring why primary care teams engage in a voluntary audit and feedback program. BMC Health Services Research. 2017 Dec 2;17:803.

Notes:

1. One interview was held with a QIDSS alone. Given that these staff work with multiple FHTs (in this case 4), these practices were excluded from this summary.
2. SAMI = Standardized Adjusted Clinical Group Morbidity Index
3. Status indicator for a Family Health Team’s access to Hospital Discharge Data.
4. ED = Executive Director
5. Physician Leader at the Family Health Team
6. Quality Improvement Decision Support Specialist
7. Interdisciplinary Health Professional (Nurse, Dietician, Social Worker, etc.)
